# Binding of Bezafibrate to Human Serum Albumin: Insight into the Non-Covalent Interaction of an Emerging Contaminant with Biomacromolecules

**DOI:** 10.3390/molecules17066821

**Published:** 2012-06-04

**Authors:** Yajie Qian, Xuefei Zhou, Jiabin Chen, Yalei Zhang

**Affiliations:** 1 Key Laboratory of Yangtze River Water Environment for Ministry of Education, College of Environmental Science and Engineering, Tongji University, Shanghai 200092, China; 2 State Key Laboratory of Pollution Control and Resources Reuse, College of Environmental Science and Engineering, Tongji University, Shanghai 200092, China

**Keywords:** bezafibrate, non-covalent interaction, fluorescence spectrometry, conformational change

## Abstract

In recent years, bezafibrate (BZF) has been frequently detected in environmental media. In order to reveal the toxicity of such an emerging pollutant, its interaction with human serum albumin (HSA) was studied by fluorescence spectrometry, circular dichroism, and equilibrium dialysis. Fluorescence data showed that the fluorescence quenching of HSA by BZF resulted from the formation of HSA-BZF complex. The binding constants were determined to be 3.33 × 10^3^, 2.84 × 10^3^ M^−1^ at 298 and 309.5 K, respectively. The thermodynamic determination indicated that the hydrophobic and electrostatic interaction were the dominant binding force. The conformational investigation showed that the presence of BZF increased the α-helix content of HSA and induced the slight unfolding of the polypeptides of protein. Finally, the equilibrium dialysis showed that 0.56 mM BZF decreased the binding of vitamin B_2_ to HSA by 29%.

## 1. Introduction

Recently, the occurrence and fate of pharmaceuticals and personal care products (PPCPs) in the environment have become a major cause for concern due to their potential toxic effect on the ecosystem and public health [1,2,3]. Bezafibrate (BZF) belongs to the group of fibrate drugs, which is an important class of pharmaceuticals widely used to decrease plasma triglycerides and raise the level of high-density lipoprotein cholesterol [4]. BZF was included in the list of most used pharmaceuticals in the World [5]. Due to its large consumption and persistence, BZF has been frequently detected in the sewage, surface, and drinking water [6,7,8,9,10]. According to Quinn *et al*. [11], BZF could be classified as harmful for non-target organisms, and it significantly affected the feeding, attachment and hydrant growth of the cnidarian *Hydra attenuata*. Acute and chronic toxicity tests performed on the rotifer *B. calyclflorus*, and crustaceans *D. magna* and *C. dubia* showed that BZF had LC_50_ and EC_50_ values within the 0.44–100 mg/L range [12]. As for humans, BZF was supposed to induce an acute renal failure for the patients treated with hyperlipemia [13].

Human serum albumin (HSA) is the most abundant protein in blood plasma, which has a number of physiological functions involving transport of various endogenous and exogenous chemicals, e.g., pharmaceuticals. The distribution, metabolism, and toxicity of such chemicals are significantly affected by their binding to HSA. Moreover, there is evidence of secondary structural change of HSA induced by its interaction with exogenous chemicals which will affect the physiological function of HSA [14]. Therefore, the interaction between HSA and pharmaceuticals are of imperative and importance. Various techniques have been used to study these interactions, e.g., fluorescence spectrometry [15], FT-IR [16], Raman spectrometry [17], circular dichroism (CD) [18], equilibrium dialysis [19], isothermal titration calorimetry [20], and capillary electrophoresis [21]. Compared with other analytical techniques, fluorescence spectrometry is a conventional and powerful method to study the molecular interactions involving proteins, owing to its sensitivity, rapidity and simpleness. From analysis of the HSA spectrum before and after the addition of ligands, a great amount of information could be obtained, such as the binding constant and mode, the microenvironment information of the fluorophore and so on [22]. In this study, the interaction between BZF and HSA was studied by fluorescence spectrometry, CD, equilibrium dialysis. Great attempts were made to investigate the binding constant and site, the binding forces, and the effect of BZF on the conformational change, and subsequent physiological function of HSA.

## 2. Results and Discussion

### 2.1. Fluorescence Quenching of HSA by BZF

**Figure 1:**
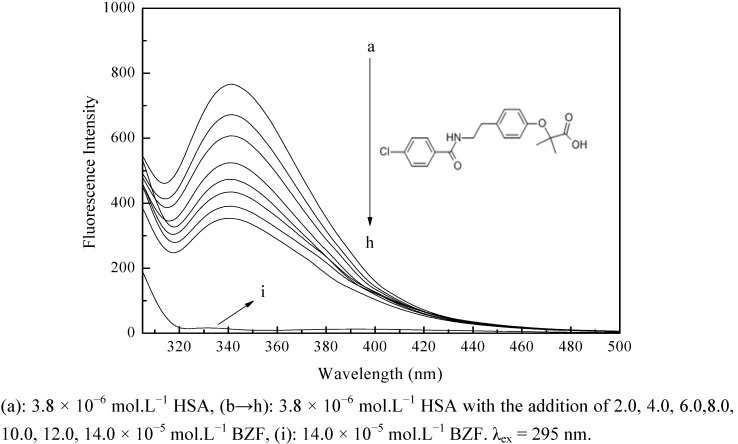
Effect of BZF on the fluorescence emission spectra of HAS.

The interaction between HSA and BZF was studied by examining the effect of BZF on the fluorescence emission spectrum of HSA. Figure 1 shows the fluorescence spectra of HSA in the presence of various concentrations of BZF. With different amounts of BZF titrated into the HSA solution, the fluorescence intensities of HSA decreased regularly with no shift of the emission wavelength, which suggested that BZF could interact with HSA and quench its intrinsic fluorescence. There was no fluorescence intensity from BZF at around λ_em_ 343 nm, thus the effect of BZF on the fluorescence intensity of HSA could be negligible.

The fluorescence quenching can be classified as either static quenching or dynamic quenching. The static quenching is induced by the formation of ground complex, while the dynamic one is initiated by the collisions between fluorophore and quencher. They can be often distinguished by their different dependence on the temperature and viscosity. The dynamic quenching constants are expected to be larger at higher temperatures because of the larger diffusion coefficients; while the static quenching constants are supposed to be smaller at higher temperatures due to the decreased stability of the complex.

**Figure 2:**
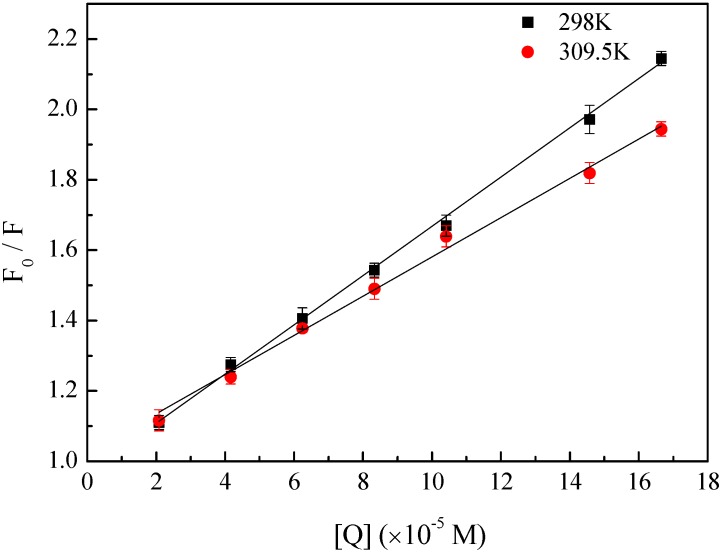
Stern-Volmer plots for the quenching of HSA by BZF.

In order to confirm the quenching mechanism, the fluorescence quenching data were analyzed with the Stern-Volmer equation:


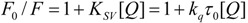
(1)

where *F_0_* and *F* were the fluorescence intensities of HSA before and after the addition of quencher. [*Q*] was the concentration of quencher, *K_SV_* the Stern-Volmer quenching constant, τ_0_ life time of the fluorophore without quencher (τ_0_ = 10^−8^ s), *k_q_* the bimolecular quenching rate constant. *F_0_*/*F* were linearly regressed with [*Q*] at two different temperatures (Figure 2), and *K_SV_* and *k_q_* were obtained from the slopes of the curves, which were displayed in Table 1. The values of *K_SV_* were inversely correlated with temperature, suggesting the fluorescence quenching of HSA was not initiated by the dynamic quenching, but the formation of ground-state complex [23]. Furthermore, the values of *k_q_* were larger than the maximum scattering collision quenching constants (2 × 10^10^ L.mol^−1^.s^−1^), which further implied the fluorescence quenching of HSA was induced by static quenching [24].

When small molecules bind independently to a set of equivalent site on a macromolecule, the binding constants (*K_a_*) and the number of binding sites (*n*) could be determined by the following equation [25]:


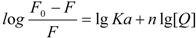
(2)

The values of *K_a_* and *n* were obtained and displayed in Table 2. The decreasing trend of *K_a_* with increasing temperature was in accordance with *K_SV_*’s dependence on temperature. HSA has one tryptophan which significantly contributes to the fluorescence of HSA, located in the hydrophobic pocket of subdomain IIA. After BZF inserted into the hydrophobic pocket, the hydrophobic part of BZF interacted with tryptophan residue, and then quenched its intrinsic fluorescence.

**Table 1:** Stern-Volmer quenching constants for the interaction between HSA and BZF.

T (K)	*K_sv_* (×10^3^ M^−1^)	*k_q_* (×10^11^ M^−1^ S^−1^)	R^2^
298	6.99	6.99	0.9985
309.5	5.58	5.58	0.9962

**Table 2:** The binding constants and thermodynamic parameters for BZF binding to HSA.

T (K)	*K_a_* (×10^3^ M^−1^)	n	R^2^	*ΔH* (kJ.mol^−1^)	*ΔG* (kJ.mol^−1^)	*ΔS* (J.mol^−1^.K)
298	3.33	0.9	0.9922	−10.54	−20.11	32.07
309.5	2.84	0.91	0.9959		−20.47	

### 2.2. Determination of the Binding Force

The acting force contributing to the interaction between macromolecule and small ligands typically include hydrogen bond, van der Waals force, electrostatic and hydrophobic interaction [26]. The thermodynamic parameters, such as enthalpy change (*ΔH*) and entropy change (*ΔS*) are always used to reveal the binding force. For this purpose, thermodynamic parameters, e.g., *ΔH*, *ΔS*, and *ΔG* were calculated based on the van’t Hoff’s equation: 


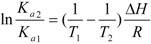
(3)

and thermodynamic equation: 


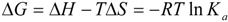
(4)

here *K_a1_* and *K_a2_* are the binding constants at *T_1_* and *T_2_*, R is the gas constant. The thermodynamic parameters of the interaction were displayed in Table 2. The negative sign of *ΔG* indicated the spontaneous process of the interaction. That *ΔH* < 0 indicated that the BZF-HSA interaction was exothermic. The absolute values of *ΔH* was much less than 250 kJ.mol^−1^, so HSA-BZF interaction was non-covalent [14]. According to Ross and Subramanian [27], the positive *ΔS* was often taken as the evidence for the hydrophobic interaction, and the negative *ΔH* and positive *ΔS* values were characteristic of specific electrostatic interactions. Therefore, both hydrophobic and electrostatic interactions might involve in the interaction. BZF is negatively charged in the neutral condition, so it would be first attracted around the alkaline amino residues of HSA by electrostatic attraction, then the BZF molecule entered the hydrophobic pocket of HSA and the hydrophobic benzene ring reacted with the non-polar amino acid residues. The combination of such non-covalent interactions might make BZF bind to HSA firmly.

### 2.3. Conformational Change of HSA

#### 2.3.1. Synchronous Fluorescence

**Figure 3:**
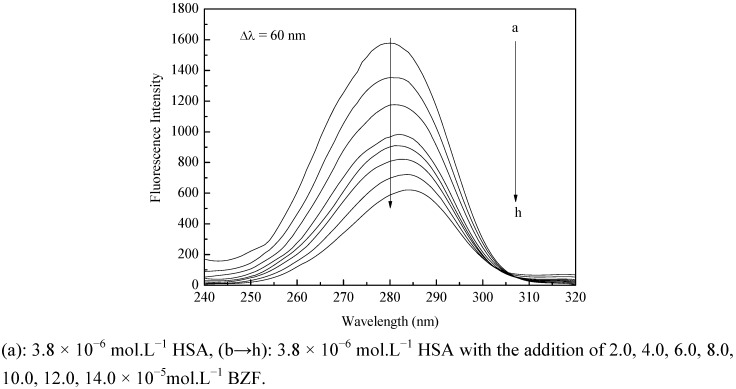
Synchronous fluorescence spectra of HAS.

Synchronous fluorescence spectrometry can provide the information about molecular environment in the vicinity of the chromophore molecules [28]. When Δλ between excitation and emission wavelength is stabilized at 60 nm, the synchronous fluorescence spectrum presents the fluorescence characteristic information of tryptophan residue. The effect of BZF on HSA synchronous fluorescence spectrum was presented in Figure 3. It could be seen from this figure that the maximum emission wavelength had a slight red shift (from 280 to 284 nm) at the investigated concentration range. The red shift indicated that the exposure degree of the tryptophan residue increased and the polarity of the microenvironments around the tryptophan residue increased.

#### 2.3.2. Three-Dimensional Fluorescence

**Figure 4:**
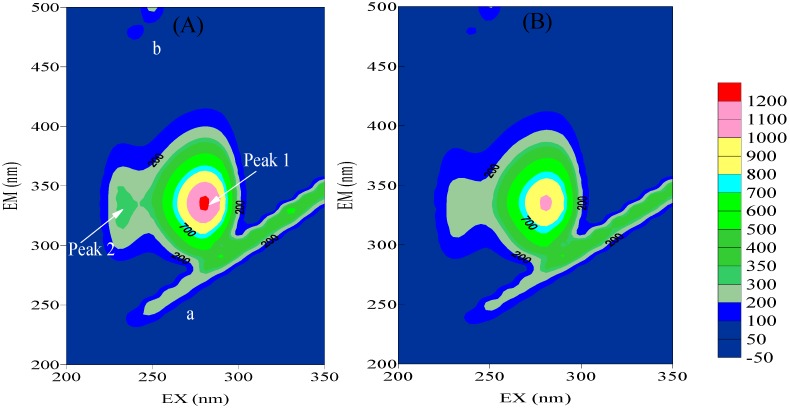
Three dimensional fluorescence spectra of HSA before and after the addition of BZF (**a**) c(HSA) =3.8 μM; (**b**) c(HSA) = c(BZF) = 3.8 μM.

Three dimensional fluorescence spectrum can present comprehensive fluorescence information about proteins, which makes the investigation of the conformational change of protein be more credible [29]. The three-dimensional fluorescence spectra of HSA before and after addition of BZF were displayed in Figure 4, and the corresponding parameters are summarized in Table 3. 

**Table 3:** Three-dimensional fluorescence spectral characteristics of HSA and HSA-BZF system.

	HSA				HSA+BZF		
	Peak position λ_ex_/λ_em_	Stokes	Intensity F		Peak position λ_ex_/λ_em_	Stokes	Intensity F
	(nm/nm)	(Δλ)			(nm/nm)	(Δλ)	
Peak 1	280/330	50	1110		280/334	54	1028
Peak 2	230/320	110	344		230/330	100	264

There were four peaks present in the spectra. Peak a represented the Rayleigh scattering peak (λ_em_ = λ_ex_); peak b was the second-order Rayleigh scattering peak (λ_em_ = 2λ_ex_). Peak 1 demonstrated the intrinsic fluorescence of tyrosine and tryptophan residues. The fluorescence intensity of peak 1 was quenched by 7.4% after the addition of BZF, and the maximum emission wavelength had a little red shift (from 330 nm to 334 nm). Peak 2 mainly exhibited the fluorescence behavior of polypeptide backbone structure, which could be attributed to the transition of P→P* of characteristic polypeptide backbone structure C=O of HSA [30]. The fluorescence intensity of peak 2 was quenched by 23.3% after interacting with BZF, indicating the unfolding of polypeptide of HSA and conformational change [31].

#### 2.3.3. CD Spectra

**Figure 5:**
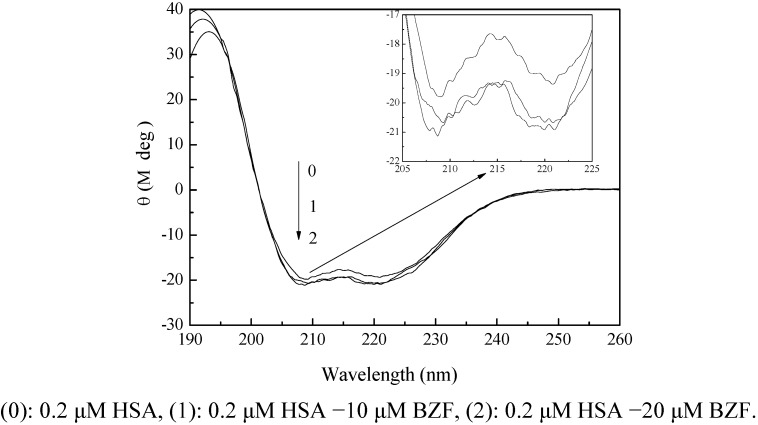
The molar ellipticity CD curves for HSA solutions before and after addition of BZF.

To further verify the structural change of HSA after the addition of BZF, the CD technique was employed because of its sensitive prediction about the contents of four secondary structures. The CD spectra of HSA in presence of various concentrations of BZF are displayed in Figure 5. The CD spectrum of HSA exhibited two negative bands in the far-UV region at 208 and 222 nm, which were characteristic of the α-helix in the protein secondary structure. After the addition of BZF, the contents of α-helix of HSA increased from 26.5% to 38.2%, whereas the fractions of β-pleated sheet and random coil decreased to various degrees. Thus the addition of BZF transformed some β-pleated sheet and random coil into α-helix. Therefore, BZF was bound to the amino acid residues of HSA by non-covalent interactions, which destroyed the hydrogen bonding network, causing the unfolding of polypeptide of HSA and the secondary structure change of HSA.

#### 2.3.4. Effect of BZF on the HSA Physiological Function

**Figure 6:**
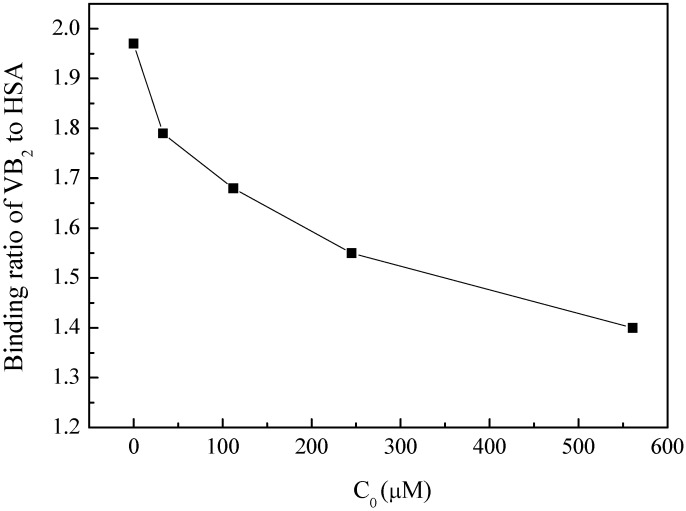
Effects of BZF on the physiological function of HSA to transport VB_2_.

The relationship between protein structural change and its physiological function is very important in organisms. A small organic compound may bind to polypeptide chain of HSA, regulating its structure and even change its physiological activity [32]. Human serum albumin is the major carrier protein in blood which can transport a large number of ligands, e.g., pharmaceuticals, vitamins and so on. The effect of BZF on HSA transportation function was determined as shown in Figure 6. With the addition of BZF, the binding ratio of VB_2_ to HSA decreased obviously compared to that without BZF, indicating the inhibition of the transportation function of HSA. 0.56 mM BZF reduced the binding ratio of VB_2_ to HSA by 29% at physiological condition. This could be attributed to the competition of the binding sites of VB_2_, and the subsequent conformation change. Therefore, non-covalent binding of such chemical affected the physiological function of protein [20].

## 3. Experimental

### 3.1. Instruments and Chemicals

The fluorescence spectrum measurement was performed on an F-4500 fluorophotometer (Hitachi, Japan) equipped with 1 cm quartz cell and a thermostat bath. CD measurement was conducted on a J-715 CD spectropolarimeter (Jasco instruments, Japan). The RC 30-5K semi-permeable membranes (Molecular Weight Cut Off 5 KDa, Shanghai Green Bird, China) were used for equilibrium dialysis. HSA, BZF and vitamin B2 (VB2) were purchased from Sigma-Aldrich (USA). They were dissolved in deionized water as a stock solution which were stored at 4 °C prior of use. A phosphate buffer (pH 7.4) was prepared by dissolving an appropriate amount of potassium dihydrogen phosphate and disodium hydrogen phosphate in deionized water.

### 3.2. Spectral Measurement of HSA–BZF Interaction

A solution (2.5 mL) containing 3.8 μM HSA was added to a quartz cell. The BZF solution was then gradually titrated to the cell using a trace syringe in concentrations ranging from 0 to 144.0 μM. The fluorescence intensities of HSA were recorded at 298 and 309.5 K, with a thermostat bath keeping the temperature constant. The widths of excitation and emission slits were set at 10 and 20 nm, respectively, and the scanning speed was at 2400 nm/min. An excitation wavelength of 295 nm was chosen and the emission wavelength was recorded from 300 to 500 nm. The synchronous fluorescence spectrum was scanned from 260 to 320 nm (Δλ = 60 nm). 

Three-dimensional fluorescence spectra were measured under the following conditions: the initial excitation wavelength was set at 200 nm with the increment of 10 nm, and the emission wavelength was recorded between 200 and 500 nm; the scan speed was set at 12,000 nm/min. Other scanning parameters were the same as those for the fluorescence emission spectra.

CD spectra were recorded at 298 K in the range of 190-260 nm with 0.1 nm data pitch. Each spectrum was the average of three successive scans with the scanning speed of 100 nm/min. The buffer solution running under the same condition was used as blank and the contribution was subtracted from the experimental spectra. The relative contents of secondary structure forms of HSA, e.g., α-helix, β-pleated sheet, β-turn and random coil, were calculated by the secondary structure estimation-standard analysis measurement software of the spectropolarimeter.

### 3.3. Effect of BZF on the Physiological Function of HSA

Effect of BZF on the physiological function of HSA to transport VB_2_ was determined by equilibrium dialysis with a special dialysis device designed by Gao’s group [20]. A solution (12.5 mL) containing phosphate buffer, 0.025 mM HSA, 0.076 mM VB_2_ and 0–0.56 mM BZF was added into the dialysis bag, which was merged in dialysate solution (37.5 mL) containing phosphate buffer. After equilibration for 10 h at 309.5 K, dialysate solution (2.5 mL) was sampled, and the fluorescence was measured with the excitation and emission wavelength at 440 and 525 nm, respectively. Thus, the concentration of VB_2_ in the dialysis solution was determined and the ratio of VB_2_ binding to HSA calculated.

## 4. Conclusions

The interaction between BZF and HSA was characterized by fluorescence spectrometry, CD and equilibrium dialysis. The quenching mechanism of fluorescence of HSA was mainly initiated by the complex formation instead of dynamic collision. Thermodynamic determination of the interaction indicated hydrophobic and electrostatic interaction dominated in the binding of BZF to HSA. The binding of BZF induced the unfolding of the polypeptides of HSA and transferred the secondary conformation of HSA. The equilibrium dialysis showed that only 0.56 mM BZF decreased vitamin B_2_ by 29% transported on the HSA, indicating the physiological function of HSA to transport VB_2_ was inhibited.
